# Impact of obesity on sentinel lymph node biopsy outcomes and survival in breast cancer patients: A single‐center retrospective study

**DOI:** 10.1002/cam4.7248

**Published:** 2024-05-11

**Authors:** Jian Pang, Lun Li, Nana Yin, Mei Dai, Shuyue Zheng, Ming Chen, Jingyan Xue, Jiong Wu

**Affiliations:** ^1^ Department of General Surgery, The Second Xiangya Hospital Central South University Changsha China; ^2^ Department of Breast Surgery, Shanghai Cancer Center Fudan University Shanghai China; ^3^ Department of Operating Room, Changde First People's Hospital Changde China; ^4^ Collaborative Innovation Center for Cancer Medicine Shanghai China

**Keywords:** early‐stage breast cancer, obesity, prognosis, retrospective study, sentinel lymph node biopsy

## Abstract

**Background:**

Sentinel lymph node biopsy (SLNB) is a common choice for axillary surgery in patients with early‐stage breast cancer (BC) who have clinically negative lymph nodes. Most research indicates that obesity is a prognostic factor for BC patients, but studies assessing its association with the rate of positive sentinel lymph nodes (SLN) and the prognosis of patients with early BC undergoing SLNB are limited.

**Methods:**

Between 2013 and 2016, 7062 early‐stage BC patients from the Shanghai Cancer Center of Fudan University were included. Based on the Chinese Body Mass Index (BMI) classification standards, the patients were divided into three groups as follows: normal weight, overweight, and obese. Propensity score matching analysis was used to balance the baseline characteristics of the participants. Logistic regression analysis was used to determine the association between obesity and positive SLN rate. Cox regression analysis was used to investigate whether obesity was an independent prognostic factor for early‐stage BC patients who had undergone SLNB.

**Results:**

No significant association was observed between obesity and positive SLN rate in early‐stage BC patients who had undergone SLNB. However, multivariate analysis revealed that compared to patients with normal BMI, the overall survival (hazard ratio (HR) 2.240, 95% confidence interval (CI) 1.27–3.95, *p* = 0.005) and disease‐free survival (HR 1.750, 95% CI 1.16–2.62, *p* = 0.007) were poorer in patients with high BMI.

**Conclusion:**

Obesity is an independent prognostic factor for early‐stage BC patients who undergo SLNB; however, it does not affect the positive SLN rate.

## 1 INTRODUCTION

The global prevalence of obesity has been steadily increasing, with an estimated 1.35 billion individuals categorized as overweight and 573 million as obese. Several factors, including eating habits, environment and sedentary lifestyle, predispose to obesity, and the absolute numbers are anticipated to increase to 2.16 billion overweight and 1.12 billion obese individuals by 2030.^1^ Obesity is a complex chronic condition that increases the risk of long‐term medical complications often associated with metabolic diseases, cardiovascular and cerebrovascular diseases, respiratory diseases, diabetes, and cancers.^2^, ^3^ This considerably increases healthcare resource utilization and medication costs.^4^

Breast cancer (BC) is the most common type of cancer in women,^5^ and obese individuals diagnosed with BC typically have unfavorable prognoses.^6^ Obesity is an independent prognostic factor for BC and a risk factor for BC recurrence and metastasis.^7^, ^8^ The European Prospective Investigation into Cancer and Nutrition study reported that every five‐unit increase in body mass index (BMI) before diagnosis increased the overall mortality risk by 10% and a 7% increase in mortality due to BC.^9^ A meta‐analysis of six studies consisting of 984 human epidermal growth factor receptor 2 (HER2)‐positive BC patients treated with neoadjuvant targeted therapy demonstrated a significant correlation between overweight/obesity and a lower pathological complete response rate.^10^ Furthermore, a high BMI was negatively correlated with chemotherapy response among patients undergoing neoadjuvant chemotherapy for BC.^11^ Additionally, a high BMI was associated with a worse prognosis in premenopausal hormone receptor‐positive BC patients receiving adjuvant endocrine therapy.^12^

Moreover, the risk of complications following breast surgery is higher among patients with obesity. Patients with a preoperative BMI ≥30 kg/m^2^ had a significantly higher risk of lymphedema than those with a preoperative BMI < 25 kg/m^2^ (odds ratio [OR] 95% confidence interval [CI] = 2.928 [1.565–5.480]).^13^ Furthermore, sentinel lymph node biopsy (SLNB)^14^ performed in overweight women was significantly associated with failed localization.^15^, ^16^, ^17^ In addition, BC patients with a high BMI have a higher rate of lymph node metastasis.^18^ However, evidence is limited for the rate of sentinel lymph node (SLN) metastasis and the prognosis following SLNB in obese patients with BC. This highlights the need for further research to better understand the implications of SLN in patients with obesity, which could help strategize tailored treatment approaches for these specific patients. Therefore, assessing the effect of obesity on the rate of positive SLN and the prognosis of patients with early‐stage BC following SLNB is imperative.

## 2 PATIENTS AND METHODS

### 2.1 Data source and study design

This retrospective, single‐center study conducted at the Shanghai Cancer Center of Fudan University initially enrolled 18,700 patients between 2013 and 2016. The patient population had BMI ranging from 12.9 kg/m^2^ to 39.13 kg/m^2^. Our study included 7062 early‐stage BC patients who had undergone SLNB. Patients who met the following criteria were included in the final analysis: (1) primary invasive BC without metastatic diseases, (2) clinical stage T_1‐2_N_0_M_0_, (3) have not undergone prior neoadjuvant therapy such as chemotherapy, radiotherapy, or endocrine therapy, (4) have no pertinent history of hospitalization or surgery, due to conditions such as hypertension, diabetes mellitus, or other underlying diseases, (5) BMI ≥18.5 kg/m^2^. SLNB is indicated in patients with early operable invasive BC, where the axillary lymph nodes were clinically assessed as negative (combination of physical examination and imaging techniques) or clinically assessed as abnormal, but the biopsy of suspicious lymph nodes was negative. SLNB is not recommended for patients with inflammatory BC, pregnant women, or those with confirmed axillary lymph node involvement based on clinical assessment and/or pathological biopsy.

### 2.2 Imaging evaluation of the axillary lymph nodes

In this study, we conducted a comprehensive imaging evaluation of the axilla using magnetic resonance imaging (MRI), ultrasound, and mammography. Our evaluation focused on various aspects of lymph nodes, including their size, shape, internal echo characteristics, and vascular conditions. On ultrasound examinations, normal lymph nodes typically appear as oval structures less than 1 cm in size with clear boundaries, uniform internal structures, and lower echogenicity. During the MRI evaluation, in addition to the size and shape of the lymph nodes, we paid particular attention to changes in MRI signals. Normal lymph nodes typically exhibit a uniform medium signal intensity on pre‐contrast imaging sequences and demonstrate uniform enhancement after contrast enhancement. A rapid enhancement in the early phase and a washout phenomenon in the delayed phase are indicative of an abnormal blood flow pattern in the lymph nodes, suggesting possible lymph node involvement. All imaging evaluations were performed by experienced radiologists.

### 2.3 Laboratory data

Clinical and pathological staging of tumors were based on the American Joint Committee on Cancer Staging Manual, 8th edition.^19^ HER2 status was assessed using immunohistochemistry (IHC), with scores of 3+ and 1+ indicating positive and negative expression, respectively. In cases where HER2 status cannot be accurately determined via IHC (score of 2+), further evaluation using fluorescence in situ hybridization is necessary. A HER2/CEP17 ratio value ≥2.0 indicates a positive expression. Estrogen receptor (ER) and progesterone receptor (PR) were considered positive if sections demonstrated at least 1%, whereas Ki67 was considered high if sections showed at least 14%.

### 2.4 Ethics, consent, and authority

This study was conducted in accordance with the Declaration of Helsinki and approved by the Ethics Committee of the Shanghai Cancer Center, Fudan University (Shanghai, China; ID: 050432–4‐1911D, 1905202–7). Informed consent was obtained from all patients during database construction.

### 2.5 Definition of obese patients

The World Health Organization guidelines for BMI classification for adults are as follows: BMI of 18.5 kg/m^2^–24.9 kg/m^2^, 25 kg/m^2^–29.9 kg/m^2^, and ≥ 30 kg/m^2^ is categorized as normal weight, overweight, and obese, respectively. This standard was established based on data from European and American populations.^20^ However, owing to differences in skeletal structure, height, and muscle development between Asians and Europeans/Americans, a reference standard was proposed for defining overweight and obesity boundary values that are more suitable for the Chinese population. Accordingly, the normal weight range is defined as 18.5 kg/m^2^ ≤ BMI < 24 kg/m^2^, 24 kg/m^2^ ≤ BMI < 28 kg/m^2^ is classified as overweight, and a BMI ≥28 kg/m^2^ is classified as obese. In this study, with reference to the Chinese BMI obesity standard, patients with a BMI of >28 kg/m^2^ were considered obese.^21^

### 2.6 Study endpoints

The primary endpoints were overall survival (OS) and disease‐free survival (DFS). OS was defined as the time from diagnosis until death from any cause, whereas DFS was defined as the time from diagnosis until tumor recurrence, metastasis, or death from any cause.

### 2.7 Data integration and statistical methods

Differences in clinical and pathological characteristics between the groups were assessed using the chi‐square test. Factors with significant differences (*p*<0.05) between the groups were incorporated into a multivariate logistic regression model for further exploration of independent factors. To minimize the impact of confounding factors (such as age, menopausal status, HER2 status, adjuvant hormone therapy, adjuvant chemotherapy, and adjuvant targeted therapy) on the research results, propensity score matching (PSM) was used to balance these confounders between the groups. The PSM method was applied to evaluate the differences in research objectives between one‐to‐one matched patient groups, which may involve excluding some data from the cohorts. Survival curves were constructed using the Kaplan–Meier (KM) method, and significance was tested using the log‐rank test. Cox proportional hazards regression analysis was used to estimate the hazard ratio (HR) for each factor with a 95% CI. Data analysis and graphical representation were performed using R software (R version 4.1.3). All statistical analyses with a *p*‐value less than 0.05 were considered statistically significant.

## 3 RESULTS

### 3.1 Baseline characteristics of the included participants

**FIGURE 1:**
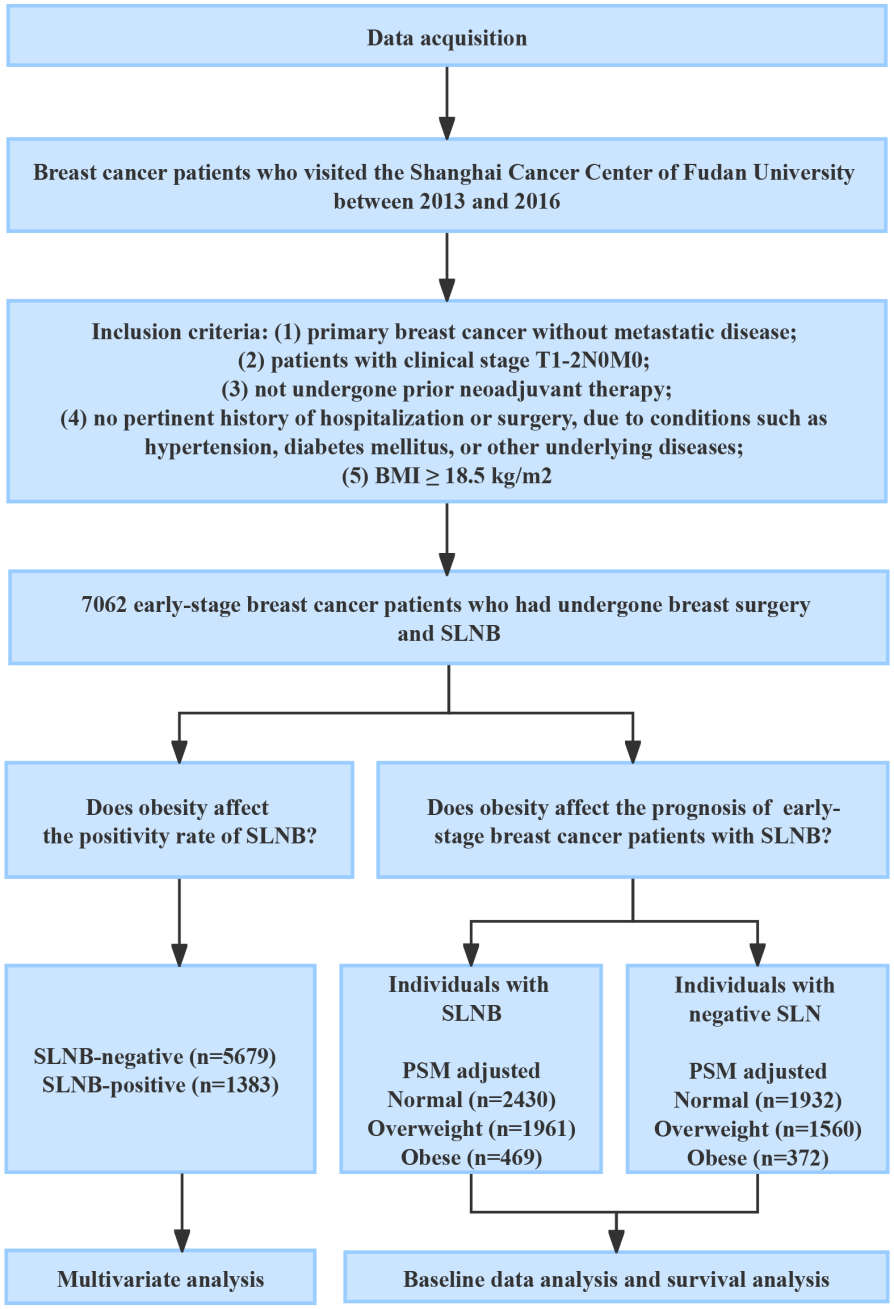
Flowchart of study design.

A flowchart of the study is shown in Figure 1. This study included 7062 early‐stage BC patients who had undergone breast surgery and SLNB, among whom lymph node metastasis was observed in 1383 (19.6%) patients. Compared with the lymph node‐negative group, the lymph node‐positive group had a higher proportion of patients under 45 years of age (14.8% vs. 11.8%) and a higher proportion of patients in the premenopausal stage (52.2% vs. 48.1%). Most patients exhibited a higher pathological T2 tumor stage (40.8% vs. 25.8%) and elevated Ki67 expression levels (78.8% vs. 74.9%). Moreover, the rates of ER positivity (82.9% vs. 72.7%), PR positivity (75.3% vs. 65.0%), and HER2 negativity (78.2% vs. 74.4%) were higher in the lymph node‐positive group (Table 1).

**TABLE 1:** Baseline characteristics of early‐stage BC patients who had undergone SLNB.

Characteristic	*N* (%)		*p*‐value
	Node‐negative (*n* = 5679)	Node‐positive (*n* = 1383)	
Age, years			
<45	668 (11.8)	205 (14.8)	**0.002**
≥45	5011 (88.2)	1178 (85.2)	
Menopausal status			
Premenopausal	2729 (48.1)	722 (52.2)	**0.006**
Postmenopausal	2950 (51.9)	661 (47.8)	
Pathologic T stage			
T1	4211 (74.2)	819 (59.2)	**<0.001**
T2	1468 (25.8)	564 (40.8)	
ER status			
Negative	1548 (27.3)	236 (17.1)	**<0.001**
Positive	4131 (72.7)	1147 (82.9)	
PR status			
Negative	1989 (35.0)	342 (24.7)	**<0.001**
Positive	3690 (65.0)	1041 (75.3)	
HER2 status			
Negative	4223 (74.4)	1082 (78.2)	**0.003**
Positive	1456 (25.6)	301 (21.8)	
Ki67(%)			
≤14	1426 (25.1)	293 (21.2)	**0.002**
>14	4253 (74.9)	1090 (78.8)	
BMI			
Normal weight	3623 (63.8)	875 (63.3)	0.862
Overweight	1643 (28.9)	410 (29.6)	
Obese	413 (7.3)	98 (7.1)	

Abbreviations: BMI, body mass index; ER, estrogen receptor; HER2, human epidermal growth factor receptor 2; T, tumor size; PR, progesterone receptor.

Bold indicates significance *p* < 0.05.

### 3.2 Factors affecting the rate of positive lymph nodes in SLNB

Multivariate analysis was conducted to investigate whether any independent factors affected the rate of positive lymph nodes, including age, menopausal status, pathologic T stage, ER status, PR status, HER2 status, subtype, and Ki67. The results indicate that age (≥ 45 vs. < 45 OR 0.811, 95% CI 0.678–0.974 *p* = 0.024), pathologic T stage (T2 vs. T1 OR 2.006, 95% CI 1.770–2.272 *p* < 0.001), ER status (Positive vs. Negative OR 1.645, 95% CI 1.291–2.092 *p* < 0.001) and Ki67 (>14% vs. ≤14% OR 1.363, 95% CI 1.174–1.587 *p* < 0.001) were independent predictive factors for SLN metastasis (Table 2).

**TABLE 2:** Independent factors affecting the rate of positive lymph nodes in early‐stage BC patients.

Characteristic	Estimate	Standard error	OR	95% CI	*p*‐value
Age, years					
<45	0.000		1.000		
≥45	−0.209	0.092	0.811	0.678–0.974	**0.024**
Menopausal status					
Premenopausal	0.000		1.000		
Postmenopausal	−0.102	0.065	0.903	0.796–1.025	0.115
Pathologic T stage					
T1	0.000		1.000		
T2	0.696	0.064	2.006	1.770–2.272	**<0.001**
ER status					
Negative	0.000		1.000		
Positive	0.498	0.123	1.645	1.291–2.092	**<0.001**
PR status					
Negative	0.000		1.000		
Positive	0.199	0.110	1.220	0.986–1.518	0.071
HER2 status					
Negative	0.000		1.000		
Positive	−0.149	0.078	0.862	0.739–1.003	0.057
Ki67(%)					
≤14	0.000		1.000		
>14	0.310	0.077	1.363	1.174–1.587	**<0.001**

Abbreviations: ER, estrogen receptor; HER2, human epidermal growth factor receptor 2; OR, odds ratio；PR, progesterone receptor; T, tumor size; 95% CI, 95% confidence interval.

Bold indicates significance *p* < 0.05.

### 3.3 Distribution characteristics of BMI in early‐stage BC patients following SLNB

The patients were classified into three groups based on the Chinese BMI criteria. Significant differences in age, menopausal status, pathologic T status, HER2 status, adjuvant hormonal therapy, adjuvant chemotherapy, and adjuvant targeted therapy were observed between the three groups. Compared to the overweight and obese groups, the proportion of normal weight group aged <45 years (15.5% vs. 6.8% vs. 7.4%, *p* < 0.001), premenopausal (55.3% vs. 39.3% vs. 30.5%, *p* < 0.001), pathologic T1 stage (74.1% vs. 66.5% vs. 65.0%, *p* < 0.001), HER2‐positive (26.1% vs. 22.9% vs. 22.1%, *p* = 0.008), receiving adjuvant chemotherapy (72.5% vs. 69.9% vs. 67.5%, *p* = 0.014), and receiving adjuvant targeted therapy (20.3% vs. 18.0% vs. 16.8%, *p* = 0.032) was higher. However, the proportion of patients receiving hormonal therapy was lower in the overweight and obese groups (73.5% vs. 75.9% vs. 76.9%, *p* = 0.044). No significant differences were observed in the distribution of SLN status, ER status, PR status, subtype and Ki67 between the different BMI groups (Table 3). After PSM, only the menopausal status remained unbalanced among the three groups, with a statistically significant difference (*p* < 0.05). However, differences in age, pathological T stage, HER2 status, adjuvant hormonal therapy, adjuvant chemotherapy, and adjuvant targeted therapy were balanced among the groups, with no statistically significant differences observed (all *p* > 0.05) (Table 3).

**TABLE 3:** Baseline distribution characteristics of BMI among early‐stage BC patients following SLNB.

Characteristic	Raw cohort, *N* (%)				Matched cohort, *N* (%)			
	Normal weight (*n* = 4498)	Overweight (*n* = 2053)	Obese (*n* = 511)	*p*‐value	Normal weight (*n* = 2430)	Overweight (*n* = 1961)	Obese (*n* = 469)	*p*‐value
Age, years								
< 45	696 (15.5)	139 (6.8)	38 (7.4)	**< 0.001**	167 (6.9)	135 (6.9)	37 (7.9)	0.716
≥ 45	3802 (84.5)	1914 (93.2)	473 (92.6)		2263 (93.1)	1826 (93.1)	432 (92.1)	
Menopausal status								
Premenopausal	2488 (55.3)	807 (39.3)	156 (30.5)	**< 0.001**	947 (39.0)	789 (40.2)	151 (32.2)	**0.006**
Postmenopausal	2010 (44.7)	1246 (60.7)	355 (69.5)		1483 (61.0)	1172 (59.8)	318 (67.8)	
Pathologic T stage								
T1	3332 (74.1)	1366 (66.5)	332 (65.0)	**< 0.001**	1666 (68.6)	1340 (68.3)	325 (69.3)	0.921
T2	1166 (25.9)	687 (33.5)	179 (35.0)		764 (31.4)	621 (31.7)	144 (30.7)	
SLN								
Negative	3623 (80.5)	1643 (80.0)	413 (80.8)	0.862	1946 (80.1)	1555 (79.3)	372 (79.3)	0.795
Positive	875 (19.5)	410 (20.0)	98 (19.2)		484 (19.9)	406 (20.7)	97 (20.7)	
ER status								
Negative	1171 (26.0)	495 (24.1)	118 (23.1)	0.127	581 (23.9)	472 (24.1)	111 (23.7)	0.981
Positive	3327 (74.0)	1558 (75.9)	393 (76.9)		1849 (76.1)	1489 (75.9)	358 (76.3)	
PR status								
Negative	1518 (33.7)	660 (32.1)	153 (29.9)	0.137	776 (31.9)	633 (32.3)	147 (31.3)	0.920
Positive	2980 (66.3)	1393 (67.9)	358 (70.1)		1654 (68.1)	1328 (67.7)	322 (68.7)	
HER2 status								
Negative	3325 (73.9)	1582 (77.1)	398 (77.9)	**0.008**	1878 (77.3)	1511 (77.1)	362 (77.2)	0.984
Positive	1173 (26.1)	471 (22.9)	113 (22.1)		552 (22.7)	450 (22.9)	107 (22.8)	
Subtype								
ER‐/PR‐/HER2‐	569 (12.7)	275 (13.4)	64 (12.5)	0.052	323 (13.3)	264 (13.5)	61 (13.0)	0.999
ER+ or PR+/HER2‐	2510 (55.8)	1190 (58.0)	304 (59.5)		1417 (58.3)	1139 (58.0)	272 (58.0)	
ER‐ and PR‐/HER2+	573 (12.7)	210 (10.2)	49 (9.6)		251 (10.3)	204 (10.4)	47 (10.0)	
ER + or PR+/HER2+	846 (18.8)	378 (18.4)	94 (18.4)		439 (18.1)	354 (18.1)	89 (19.0)	
Ki67(%)								
≤14	1106 (24.6)	502 (24.5)	111 (21.7)	0.356	580 (23.9)	473 (24.1)	101 (21.5)	0.487
>14	3392 (75.4)	1551 (75.5)	400 (78.3)		1850 (76.1)	1488 (75.9)	368 (78.5)	
Adjuvant hormonal therapy								
No	1193 (26.5)	494 (24.1)	118 (23.1)	**0.044**	582 (24.0)	475 (24.2)	115 (24.5)	0.956
Yes	3305 (73.5)	1559 (75.9)	393 (76.9)		1848 (76.0)	1486 (75.8)	354 (75.5)	
Adjuvant chemotherapy								
No	1237 (27.5)	617 (30.1)	166 (32.5)	**0.014**	733 (30.2)	585 (29.8)	150 (32.0)	0.659
Yes	3261 (72.5)	1436 (69.9)	345 (67.5)		1697 (69.8)	1376 (70.2)	319 (68.0)	
Adjuvant targeted therapy								
No	3586 (79.7)	1683 (82.0)	425 (83.2)	**0.032**	2001 (82.3)	1612 (82.2)	389 (82.9)	0.931
Yes	912 (20.3)	370 (18.0)	86 (16.8)		429 (17.7)	349 (17.8)	80 (17.1)	

Abbreviations: BMI, body mass index; ER, estrogen receptor; HER2, human epidermal growth factor receptor 2; PR, progesterone receptor; SLN, sentinel lymph node; T, tumor size.

Bold indicates significance *p* < 0.05.

### 3.4 Survival analysis in early‐stage BC patients following SLNB

The median follow‐up time for OS and DFS was 54 months (interquartile range [IQR] of 41–69 months). The number of mortalities in the normal weight, overweight, and obese groups was 75 (1.7%), 50 (2.4%), and 21 (4.1%), respectively. Local recurrence or distant metastasis were reported in 108 (2.4%), 42 (2.0%), and 15 (2.9%) patients in the normal‐weight, overweight, and obese groups, respectively.

**FIGURE 2:**
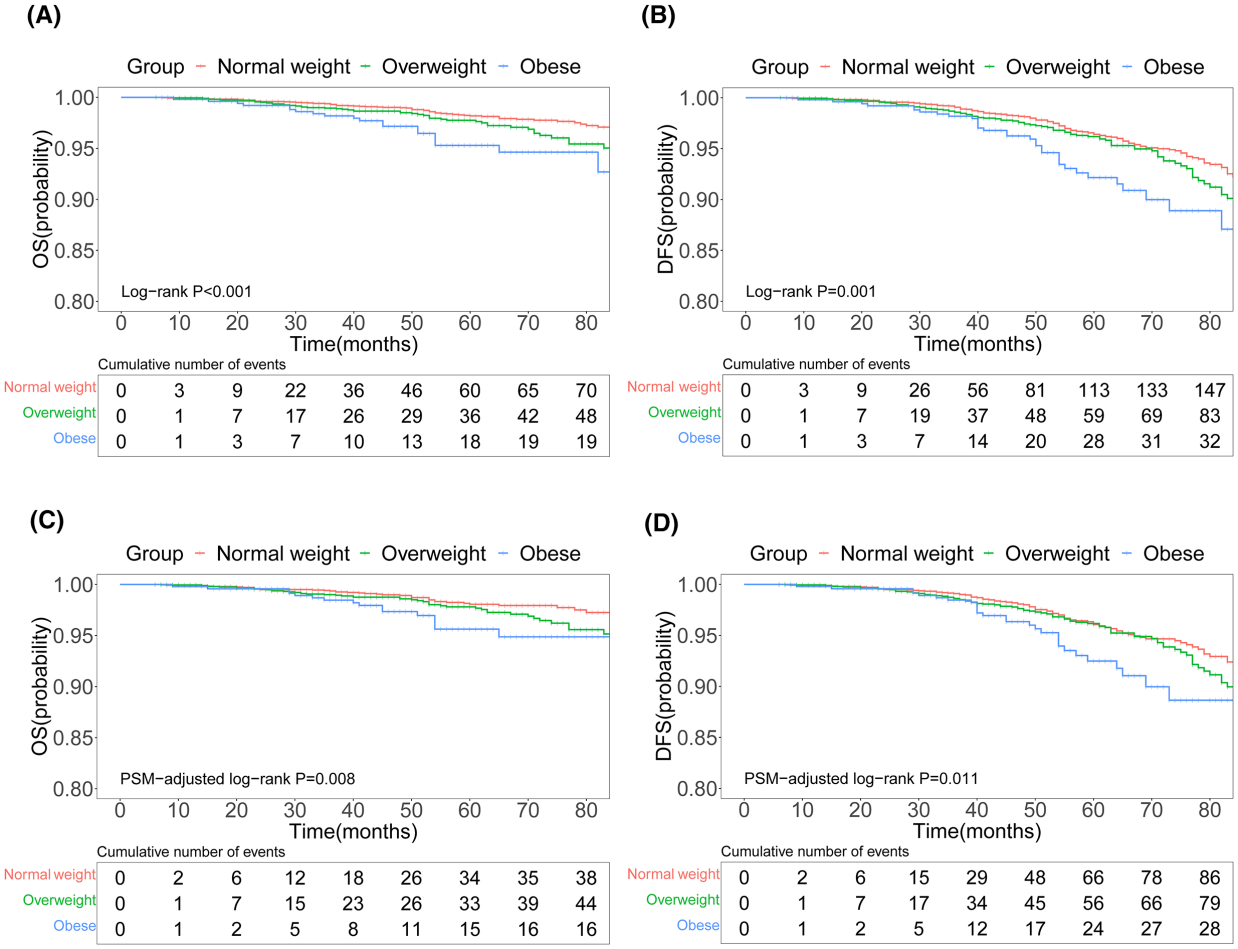
Pre‐ and post‐PSM survival curves for OS and DFS in early‐stage BC patients with SLNB. DFS, disease‐free survival; OS, overall survival; PSM, propensity score matching.

In the original cohort, the OS rates of the normal weight group at 36, 60, and 84 months were 99.3%, 98.2%, and 97.1%, respectively, all of which were higher than those of the overweight (98.9%, 97.8%, and 95.0%, respectively) and obese groups (98.2%, 95.3%, and 92.7%, respectively) (*p* < 0.001) (Figure 2A). The DFS rates at 36, 60, and 84 months of the normal weight group were 99.1%, 96.4%, and 92.2%, respectively, which were higher than those of the overweight (98.6%, 96.2%, and 90.1%, respectively) and obese groups (98.2%, 92.2%, and 87.1%, respectively) (*p* = 0.001) (Figure 2B). PSM results revealed that overweight or obese patients had reduced survival benefits in terms of both OS and DFS (*p* = 0.008 and *p* = 0.011, respectively) (Figure 2C,D).

### 3.5 Factors influencing the prognosis of early‐stage BC patients following SLNB

Multivariate analysis performed after PSM revealed that menopausal status (postmenopausal vs. premenopausal: HR 2.490, 95% CI 1.54–4.04, *p* < 0.001), pathologic T stage (T2 vs. T1: HR 2.100, 95% CI 1.43–3.09, *p* < 0.001) and BMI (obese vs. normal weight: HR 2.240, 95% CI 1.27–3.95, *p* = 0.005) were independent risk factors for OS (Table 4). Additionally, menopausal status (postmenopausal vs. premenopausal: HR 1.570, 95% CI 1.16–2.12, P = 0.003), pathologic T stage (T2 vs. T1: HR 1.950, 95% CI 1.47–2.57, *p* < 0.001), SLN (positive vs. negative: HR 1.650, 95% CI 1.21–2.24, *p* = 0.001), PR status (positive vs. negative: HR 0.580, 95% CI 0.37–0.89, *p* = 0.013), Ki67 (>14% vs. ≤14%: HR 1.510, 95% CI 1.04–2.19, *P* = 0.030), and BMI (obese vs. normal weight: HR 1.750, 95% CI 1.16–2.62, *p* = 0.007) were independent risk factors for DFS (Table S1).

**TABLE 4:** Independent factor analysis of OS rates in patients with early stage BC after PSM.

Factor	Univariate analysis			Multivariate analysis		
	HR	95% CI	*p*‐value	HR	95% CI	*p‐*value
Age, years (≥ 45 vs. < 45)	1.050	0.49–2.26	0.904			
Menopausal status (postmenopausal vs. premenopausal)	2.470	1.53–3.98	**< 0.001**	2.490	1.54–4.04	**< 0.001**
Pathologic T stage (T2 vs. T1)	2.100	1.43–3.08	**< 0.001**	2.100	1.43–3.09	**< 0.001**
SLN (positive vs. negative)	1.400	0.90–2.16	0.136			
ER status (positive vs. negative)	0.530	0.35–0.79	**0.002**	0.750	0.19–2.90	0.676
PR status (positive vs. negative)	0.520	0.35–0.76	**0.001**	0.670	0.35–1.28	0.224
HER2 status (positive vs. negative)	0.720	0.43–1.20	0.209			
Ki67 (>14% vs. ≤14%)	1.150	0.73–1.79	0.551			
Adjuvant hormonal therapy (yes vs. no)	0.540	0.36–0.80	**0.002**	1.090	0.29–4.00	0.901
Adjuvant chemotherapy (yes vs. no)	1.090	0.71–1.68	0.683			
Adjuvant targeted therapy (yes vs. no)	0.710	0.40–1.27	0.253			
BMI (overweight vs. normal weight)	1.440	0.94–2.19	0.092	1.470	0.96–2.24	0.075
BMI (obese vs. normal weight)	2.370	1.35–4.18	**0.003**	2.240	1.27–3.95	**0.005**

Abbreviations: BMI, body mass index; ER, estrogen receptor; HER2, human epidermal growth factor receptor 2; PR, progesterone receptor; SLN, sentinel lymph node; T, tumor size.

Bold indicates significance *p* < 0.05.

### 3.6 Distribution of BMI in early‐stage BC patients with negative SLN

To further demonstrate the prognostic value of obesity in early‐stage BC patients, we summarized the population distribution of BMI among all patients with negative SLNB results at baseline. The findings indicated statistically significant differences in age, menopausal status, pathologic T stage, HER2 status, and adjuvant targeted therapy among the different BMI groups (all *p* < 0.05). Compared to the overweight and obese groups, the proportion of patients aged <45 years (14.8% vs. 6.2% vs. 6.8%, *p* < 0.001), premenopausal (54.6% vs. 38.3% vs. 29.3%, *p* < 0.001), pathologic T1 stage (76.9% vs. 70.0% vs. 66.8%, *p* < 0.001), HER2‐positive (27.0% vs. 23.0% vs. 24.0%, *p* = 0.006), and patients receiving adjuvant targeted therapy (20.0% vs. 17.2% vs. 17.9%, *p* = 0.048) were higher in the normal weight group. No significant differences were observed in the distribution of ER status, PR status, subtype, Ki67, adjuvant hormonal therapy, or adjuvant chemotherapy among different BMI groups (Table 5). Following PSM, only the menopausal status remained unbalanced among the three groups, which was statistically significant (P < 0.05). However, differences in age, pathological T stage, HER2 status, adjuvant hormonal therapy, adjuvant chemotherapy, and adjuvant targeted therapy were balanced among the groups, with no statistically significant differences (all *p* > 0.05) (Table 5).

**TABLE 5:** Population distribution characteristics of BMI in early‐stage BC patients with negative SLN.

Characteristic	Raw cohort, *N* (%)			*p*‐value	Matched cohort, *N* (%)			*p*‐value
	Normal weight (*n* = 3623)	Overweight (*n* = 1643)	Obese (*n* = 413)		Normal weight (*n* = 1932)	Overweight (*n* = 1560)	Obese (n = 372)	
Age, years								
< 45	538 (14.8)	102 (6.2)	28 (6.8)	**< 0.001**	122 (6.3)	97 (6.2)	26 (7.0)	0.858
≥ 45	3085 (85.2)	1541 (93.8)	385 (93.2)		1810 (93.7)	1463 (93.8)	346 (93.0)	
Menopausal status								
Premenopausal	1979 (54.6)	629 (38.3)	121 (29.3)	**< 0.001**	732 (37.9)	609 (39.0)	116 (31.2)	**0.019**
Postmenopausal	1644 (45.4)	1014 (61.7)	292 (70.7)		1200 (62.1)	951 (61.0)	256 (68.8)	
Pathologic T stage								
T1	2785 (76.9)	1150 (70.0)	276 (66.8)	**<0.001**	1390 (71.9)	1128 (72.3)	269 (72.3)	0.969
T2	838 (23.1)	493 (30.0)	137 (33.2)		542 (28.1)	432 (27.7)	103 (27.7)	
ER status								
Negative	1011 (27.9)	435 (26.5)	102 (24.7)	0.267	504 (26.1)	417 (26.7)	92 (24.7)	0.721
Positive	2612 (72.1)	1208 (73.5)	311 (75.3)		1428 (73.9)	1143 (73.3)	280 (75.3)	
PR status								
Negative	1289 (35.6)	567 (34.5)	133 (32.2)	0.346	657 (34.0)	540 (34.6)	122 (32.8)	0.790
Positive	2334 (64.4)	1076 (65.5)	280 (67.8)		1275 (66.0)	1020 (65.4)	250 (67.2)	
HER2 status								
Negative	2644 (73.0)	1265 (77.0)	314 (76.0)	**0.006**	1486 (76.9)	1206 (77.3)	279 (75.0)	0.637
Positive	979 (27.0)	378 (23.0)	99 (24.0)		446 (23.1)	354 (22.7)	93 (25.0)	
Subtype								
ER‐/PR‐/HER2‐	492 (13.6)	247 (15.0)	54 (13.1)	0.053	286 (14.8)	241 (15.4)	48 (12.9)	0.735
ER+ or PR+/HER2‐	1952 (53.9)	923 (56.2)	234 (56.6)		1084 (56.1)	878 (56.3)	206 (55.4)	
ER‐ and PR‐/HER2+	493 (13.6)	180 (11.0)	43 (10.4)		213 (11.0)	174 (11.2)	41 (11.0)	
ER+ or PR+/HER2+	686 (18.9)	293 (17.8)	82 (19.9)		349 (18.1)	267 (17.1)	77 (20.7)	
Ki67(%)								
≤14	918 (25.3)	419 (25.5)	89 (21.5)	0.221	485 (25.1)	401 (25.7)	81 (21.8)	0.288
>14	2705 (74.7)	1224 (74.5)	324 (78.5)		1447 (74.9)	1159 (74.3)	291 (78.2)	
Adjuvant hormonal therapy								
No	1022 (28.2)	430 (26.2)	102 (24.7)	0.139	501 (25.9)	413 (26.5)	94 (25.3)	0.872
Yes	2601 (71.8)	1213 (73.8)	311 (75.3)		1431 (74.1)	1147 (73.5)	278 (74.7)	
Adjuvant chemotherapy								
No	1159 (32.0)	571 (34.8)	145 (35.1)	0.091	674 (34.9)	544 (34.9)	129 (34.7)	0.997
Yes	2464 (68.0)	1072 (65.2)	268 (64.9)		1258 (65.1)	1016 (65.1)	243 (65.3)	
Adjuvant targeted therapy								
No	2900 (80.0)	1361 (82.8)	339 (82.1)	**0.048**	1602 (82.9)	1295 (83.0)	302 (81.2)	0.687
Yes	723 (20.0)	282 (17.2)	74 (17.9)		330 (17.1)	265 (17.0)	70 (18.8)	

Abbreviations: BMI, body mass index; ER, estrogen receptor; HER2, human epidermal growth factor receptor 2; PR, progesterone receptor; T, tumor size.

Bold indicates significance *p* < 0.05.

### 3.7 Survival analysis of early‐stage BC patients with negative SLN

The median follow‐up time for OS and DFS was 54 months (IQR of 41–69 months). The number of reported mortalities in the normal weight, overweight, and obese groups was 52 (1.4%), 36 (2.2%), and 16 (3.9%), respectively. Local recurrence or distant metastasis were reported in 68 (1.9%), 27 (1.6%), and 13 (3.1%) patients in the normal weight, overweight, and obese groups, respectively.

**FIGURE 3:**
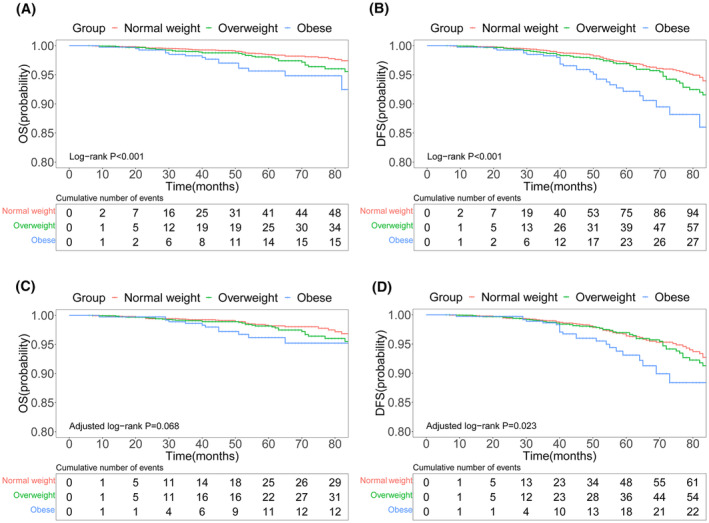
Survival curves for OS and DFS in early‐stage BC patients with negative SLN and different BMI statuses before and after PSM. BMI, body mass index; DFS, disease‐free survival; OS, overall survival.

In the original cohort, the OS rates of the normal weight group at 36, 60, and 84 months of follow‐up were 99.4%, 98.5%, and 97.5%, respectively, all of which were higher than those of the overweight (99.0%, 98.1%, and 95.6%, respectively) and obese groups (98.3%, 95.7%, and 92.5%, respectively) (*p* < 0.001) (Figure 3A). Additionally, the DFS rates of the normal‐weight group were 99.2%, 97.0%, and 93.3%, respectively, which were higher than those of the overweight group (98.7%, 96.9%, and 91.6%, respectively) and the obese group (98.3%, 92.2%, and 86.0%, respectively) (*p* < 0.001) (Figure 3B). Compared with the normal weight group, the survival benefit in terms of DFS following PSM was reduced in both overweight and obese patients (*p* = 0.023) (Figure 3D). In terms of OS, although the overall trend reflected a better prognosis on the survival curve for the normal weight group than that of the overweight and obese groups, the difference was not statistically significant (P = 0.068) (Figure 3C).

### 3.8 Factors influencing the prognosis of early‐stage BC patients with negative SLN

Multivariate cox analysis performed after PSM revealed that menopausal status (postmenopausal vs. premenopausal: HR 3.680, 95% CI 1.94–6.99, *p* < 0.001), pathologic T stage (T2 vs. T1: HR 1.750, 95% CI 1.10–2.77, *p* = 0.017) and BMI (obese vs. normal weight: HR 1.990, 95% CI 1.02–3.87, *p* = 0.043) were independent risk factors for OS in early‐stage BC patients with negative SLNB (Table 6). Furthermore, menopausal status (postmenopausal vs. premenopausal: HR 2.060, 95% CI 1.41–3.00, *p* < 0.001), pathologic T stage (T2 vs. T1: HR 1.740, 95% CI 1.26–2.40, P = 0.001), Ki67 (>14% vs. ≤14%: HR 1.550, 95% CI 1.01–2.37, *p* = 0.043), and BMI (obese vs. normal weight: HR 1.740, 95% CI 1.08–2.79, *p* = 0.022) were independent risk factors for DFS in early‐stage BC patients with negative SLN (Table S2).

**TABLE 6:** Independent factor analysis of OS rates in early‐stage BC patients with negative SLN after PSM.

Factor	Univariate analysis			Multivariate analysis		
	HR	95% CI	*p*‐value	HR	95% CI	*p*‐value
Age, years (≥ 45 vs. < 45)	1.230	0.45–3.36	0.688			
Menopausal status (postmenopausal vs. premenopausal)	3.600	1.90–6.81	**< 0.001**	3.680	1.94–6.99	**< 0.001**
Pathologic T stage (T2 vs. T1)	1.720	1.09–2.71	**0.020**	1.750	1.10–2.77	**0.017**
ER status (positive vs. negative)	0.540	0.34–0.86	**0.009**	1.240	0.27–5.67	0.785
PR status (positive vs. negative)	0.560	0.36–0.87	**0.011**	0.850	0.37–1.93	0.695
HER2 status (positive vs. negative)	0.570	0.30–1.07	0.081			
Ki67 (>14% vs. ≤14%)	1.290	0.76–2.17	0.346			
Adjuvant hormonal therapy (yes vs. no)	0.520	0.33–0.82	**0.005**	0.520	0.13–2.15	0.370
Adjuvant chemotherapy (yes vs. no)	1.160	0.72–1.88	0.549			
Adjuvant targeted therapy (yes vs. no)	0.520	0.24–1.13	0.099			
BMI (overweight vs. normal weight)	1.310	0.80–2.13	0.279	1.350	0.83–2.19	0.232
BMI (obese vs. normal weight)	2.160	1.11–4.19	**0.023**	1.990	1.02–3.87	**0.043**

Abbreviations: BMI, body mass index; ER, estrogen receptor; HER2, human epidermal growth factor receptor 2; PR, progesterone receptor; T, tumor size.

Bold indicates significance *p* < 0.05.

## 4 DISCUSSION

The prevalence of obesity has been rapidly increasing in recent years.^14^ Obesity contributes to cancer progression and unfavorable survival outcomes in BC patients.^15^, ^16^ However, available data on the effect of obesity on the prognosis of early‐stage BC patients undergoing SLNB, particularly in East Asian populations, are scarce. Our findings found that obesity is an independent risk factor for prognosis in early‐stage BC patients who had undergone SLNB; however, obesity did not affect the positive SLN rate. Age, tumor stage, ER status and Ki67 expression levels were independent factors influencing SLN metastasis.

Obesity is a potential prognostic risk factor for BC patients. Chan et al. systematically reviewed 210,000 BC patients and reported 41,477 deaths, among which 23,182 were due to BC.^22^ Higher BMI was consistently correlated with lower OS and BC‐specific survival rates in BC survivors. Compared with women with normal weight, the risk of total mortality in obese women increased by 41%, 23%, and 21% before BC diagnosis, within 12 months after diagnosis, and after 12 months, respectively,^22^ concurrent increase of 35%, 25%, and 68% in BC‐specific mortality rates.^22^ Furthermore, mortality risk, recurrence, and metastasis are higher in patients with a high BMI in early‐stage BC. Marianne et al. analyzed 53,816 early‐stage BC patients and reported that individuals with a BMI > 30 kg/m^2^ had a significantly increased risk of distant metastasis (46%) and death (38%) after 10 years than those with a BMI < 25 kg/m.^18^ Moreover, Carmen et al. demonstrated that a high BMI (≥ 33.2 kg/m^2^) was associated with higher recurrence rates, second primary tumors, and mortality rates 10 years after diagnosis among of 154 early‐stage invasive BC patients.^23^ Laura et al. reported that premenopausal women who were overweight (25 kg/m^2^ ≤ BMI ≤29.9 kg/m^2^) and obese (BMI **≥**30 kg/m^2^) had higher risks of local recurrence in 878 early‐stage invasive BC patients who underwent breast‐conserving surgery.^24^

However, some studies have reported contradictory results. Grazia Vernaci et al. found that a BMI of ≥30 kg/m^2^ was not an independent factor for DFS and OS among 992 patients with early‐stage BC.^25^ Modi et al. demonstrated that among 5099 HER2+ early‐stage BC patients, a higher BMI was associated with worse OS and DFS. However, among 3496 HER2+ late‐stage BC patients, a higher BMI was significantly associated with improved OS and progression‐free survival.^26^ Several factors might contribute to these conflicting results. First, there are variations in the inclusion and exclusion criteria across different studies, as well as disparities in the definitions of overweight and obesity based on BMI. Second, a lack of balance in clinical baseline characteristics in some studies might have contributed to bias.

A more comprehensive explanation is as follows: First, environmental factors, lifestyle choices, and particularly dietary habits can affect the biological properties of adipose tissue and influence prognostic behavior.^27^, ^28^ Second, obese patients are more likely to receive reduced chemotherapy doses than those of normal weight women.^29^ In routine clinical practice, many healthcare professionals “constrain” the chemotherapy dose for obese patients to mitigate concerns about toxicity.^30^, ^31^ Suboptimal dosing in obese BC patients can result in lower survival rates in those undergoing systemic adjuvant chemotherapy.^32^ Owing to the non‐availability of specific outcomes for this data subset, robust subgroup analysis could not be performed.

Furthermore, data analysis from a Phase III randomized controlled trial (E5103) revealed a significant correlation between elevated BMI and poorer BC prognosis in women of African ancestry, whereas no such correlation was observed in women of European ancestry.^33^ However, the study highlighted that obesity‐related comorbidities such as diabetes and its complications occur at higher rates among black patients than among white patients.^33^ Additionally, disparities in socioeconomic factors may hinder access to quality care for black patients.^33^ Collectively, these factors may explain the differential impact of severe obesity on BC prognosis across different racial groups. Moreover, BMI may not accurately reflect body composition,^34^ which varies greatly among ethnicities. In contrast to BMI, muscle mass and fat mass exhibit a more pronounced association with survival outcomes in early‐stage BC.^35^ Therefore, future studies should revealing the relationship between body composition and race, as this could potentially provide more consistent and precise results.

Obesity is often accompanied by dyslipidemia and hypertension, which are potential cardiovascular risk factors. The adverse impact of elevated BMI on long‐term prognostic outcomes may be partially mediated by obesity‐related metabolic abnormalities.^36^ A meta‐analysis indicated that the presence of metabolic abnormalities is associated with an increased risk of BC in adult women, as observed in studies with a follow‐up duration of at least 10 years.^37^ However, whether the decreased survival rates observed in severely obese BC patients are a direct consequence of obesity itself or a result of obesity‐related comorbidities requires further investigation.

Interestingly, clinical trials have specifically investigated the prognosis of patients with obesity after weight‐loss interventions. For instance, the Women's Intervention Nutrition Study conducted a randomized controlled trial involving 2437 early‐stage BC patients. These patients were randomly assigned to either the reduced‐fat diet group or the control group within the first year after diagnosis. The intervention group achieved an approximate 3.2% relative weight loss.^38^ After a median follow‐up of 5 years, the intervention group demonstrated an improved DFS (HR 0.76, 95% CI 0.60–0.98). Patients with ER‐negative tumors (HR 0.58, 95% CI 0.37–0.91) and those with a baseline BMI exceeding 30 kg/m^2^ (HR 0.66, 95% CI 0.41–1.0)^38^ exhibited significant benefits. These results were consistent with our findings, indirectly supporting the reliability of our results. Although considerable progress has been made in understanding the correlation between obesity and prognostic outcomes in early‐stage BC, a direct causal relationship has not been elucidated, warranting further investigation.

All early‐stage BC patients who had undergone SLNB were included in our analysis, and PSM was performed to eliminate confounding factors. The results indicated that obesity was an independent risk factor for OS and DFS of BC patients who had undergone SLNB. In addition, a separate analysis of independent prognostic factors in early‐stage BC patients with negative SLN was performed, which demonstrated similar results. Therefore, we believe that obesity is a risk factor for early‐stage BC patients who have undergone SLNB. Compared with patients in the normal weight group, obesity did not affect the positive SLN rate. Anna et al. discovered that increasing age and BMI are not contraindications for SLNB.^39^ However, increased body weight and age are potential risk factors for surgical failure. This implies that obesity does not affect SLNB accuracy but increases the difficulty and risk of surgery.^39^ Marybeth et al. found that obesity does not significantly affect the detection rate or false‐negative rate of SLNB. They found that performing SLNB in patients with obesity did not increase the difficulty or duration of surgery.^40^ There are indeed differences in the surgical habits and techniques of surgeons, especially when performing related surgeries in patients with obesity. Based on our clinical experience, using a single dye, such as methylene blue, is often associated with a high probability of SLNB failure. The use of two dyes, methylene blue and nuclides may increase the success rate of SLNB.

This was a large retrospective cohort study intended to investigate the association between obesity and the positive SLN rate, and the prognosis in Asian patients with early‐stage BC after SLNB. The baseline characteristics were balanced to ensure that the results were reliable. However, our study had some limitations. First, as this was a retrospective study, we could not exclude the effects of confounding factors. Second, PSM may result in the loss of a certain number of patients to follow‐up, thereby reducing the clinical sample size. Third, the cohort used in our study was Chinese, and we used BMI grouping criteria appropriate for the Chinese population. Fourth, our data lacked information on obesity‐related comorbidity such as diabetes and hypertension. Obese patients often have comorbidities such as diabetes and hypertension, which may affect treatment choices and patient prognosis. Finally, we did not perform subgroup analyses of other dimensions, such as cholesterol levels, which could provide a more refined definition of obesity. Therefore, future studies with diverse population and ethnicities are warranted to increase the robustness of the association.

## 5 CONCLUSION

Overall, obesity is an independent factor for the prognosis of early‐stage BC patients who have undergone SLNB; however, it does not affect SLN metastasis.

## AUTHOR CONTRIBUTIONS

**Jian Pang:** Conceptualization (equal); formal analysis (equal); investigation (equal); methodology (equal); software (equal); validation (equal); writing – original draft (equal); writing – review and editing (equal). **Lun Li:** Conceptualization (equal); data curation (equal); formal analysis (equal); investigation (equal); methodology (equal); resources (equal); software (equal); writing – original draft (equal); writing – review and editing (equal). **Nana Yin:** Conceptualization (equal); formal analysis (equal); writing – original draft (equal); writing – review and editing (equal). **Mei Dai:** Conceptualization (equal); formal analysis (equal); software (equal); visualization (equal). **Shuyue Zheng:** Conceptualization (equal); data curation (equal); formal analysis (equal); software (equal); writing – original draft (equal). **Ming Chen:** Conceptualization (equal); data curation (equal); funding acquisition (equal); resources (equal); writing – original draft (equal). **Jingyan Xue:** Conceptualization (equal); data curation (equal); funding acquisition (equal); investigation (equal); methodology (equal); resources (equal); software (equal); validation (equal); writing – review and editing (equal). **Jiong Wu:** Conceptualization (equal); data curation (equal); investigation (equal); methodology (equal); project administration (equal); resources (equal); validation (equal); visualization (equal); writing – original draft (equal).

## FUNDING INFORMATION

This study was funded by the Academic Leaders of the Shanghai Science and Technology Commission (18XD1401300), the Youth Program of the National Natural Science Foundation of China (82002797)，the Changsha Natural Science Fundation (kq2208336), the Hunan Provincial Natural Science Fundation (2023JJ40831; 2023JJ60441), the Scientific Research Launch Project for new employees of the Second Xiangya Hospital of Central South University (2022‐086) and the Science and Technology Innovation Program of Hunan Province (2020SK53410).

## CONFLICT OF INTEREST STATEMENT

The authors have no potential conflicts of interest to declare.

## Supporting information


Table S1.

Table S2.

## Data Availability

Data supporting the findings of this study are available from the corresponding author, WJ, upon reasonable request. The data are not publicly available because they could compromise the privacy of the research participants.
