# Supplementary material for: Impact of obesity on sentinel lymph node biopsy outcomes and survival in breast cancer patients: A single‐center retrospective study
Source: Cancer Med. 2024 May 11;13(9):e7248. doi: 10.1002/cam4.7248 (PMC11087846; doi:10.1002/cam4.7248)
Supplement: Supplementary file 1 — Table S1. [file CAM4-13-e7248-s001.doc]

Supplementary Table 1

Independent factor analysis of DFS rates in patients with early stage BC after PSM

| Factor | Univariate analysis | | | Multivariate analysis | | |
| --- | --- | --- | --- | --- | --- | --- |
|  | HR | 95% CI | P-value | HR | 95% CI | P-value |
| Age, years (≥ 45 vs. < 45) | 0.800 | 0.50–1.28 | 0.358 |  |  |  |
| Menopausal status (postmenopausal vs. premenopausal) | 1.600 | 1.19–2.15 | **0.002** | 1.570 | 1.16–2.12 | **0.003** |
| Pathologic T stage (T2 vs. T1) | 2.110 | 1.62–2.75 | **< 0.001** | 1.950 | 1.47–2.57 | **< 0.001** |
| SLN (positive vs. negative) | 1.660 | 1.24–2.22 | **0.001** | 1.650 | 1.21–2.24 | **0.001** |
| ER status (positive vs. negative) | 0.550 | 0.42–0.73 | **< 0.001** | 0.730 | 0.29–1.81 | 0.495 |
| PR status (positive vs. negative) | 0.520 | 0.40–0.68 | **< 0.001** | 0.580 | 0.37–0.89 | **0.013** |
| HER2 status (positive vs. negative) | 0.920 | 0.67–1.28 | 0.635 |  |  |  |
| Ki67% (>14 vs. ≤14) | 1.880 | 1.33–2.65 | **< 0.001** | 1.510 | 1.04–2.19 | **0.030** |
| Adjuvant hormonal therapy (yes vs. no) | 0.570 | 0.43–0.76 | **< 0.001** | 1.310 | 0.54–3.19 | 0.545 |
| Adjuvant chemotherapy (yes vs. no) | 1.390 | 1.02–1.91 | **0.039** | 0.820 | 0.57–1.17 | 0.272 |
| Adjuvant targeted therapy (yes vs. no) | 1.050 | 0.74–1.49 | 0.772 |  |  |  |
| BMI (overweight vs. normal weight) | 1.120 | 0.84–1.49 | 0.431 | 1.130 | 0.85–1.51 | 0.401 |
| BMI (obese vs. normal weight) | 1.830 | 1.23–2.75 | **0.003** | 1.750 | 1.16–2.62 | **0.007** |

Abbreviations: T, tumor size; SLN, sentinel lymph node; ER, estrogen receptor; PR, progesterone receptor; HER2, human epidermal growth factor receptor 2; BMI, body mass index
