# Supplementary material for: Impact of obesity on sentinel lymph node biopsy outcomes and survival in breast cancer patients: A single‐center retrospective study
Source: Cancer Med. 2024 May 11;13(9):e7248. doi: 10.1002/cam4.7248 (PMC11087846; doi:10.1002/cam4.7248)
Supplement: Supplementary file 2 — Table S2. [file CAM4-13-e7248-s002.doc]

Supplementary Table 2 Independent factor analysis of DFS rates in early-stage BC patients with negative SLN after PSM

| Factor | Univariate analysis | | | Multivariate analysis | | |
| --- | --- | --- | --- | --- | --- | --- |
|  | HR | 95% CI | P-value | HR | 95% CI | P-value |
| Age, years (≥ 45 vs. < 45) | 1.100 | 0.56–2.16 | 0.782 |  |  |  |
| Menopausal status (postmenopausal vs. premenopausal) | 2.030 | 1.40–2.95 | **< 0.001** | 2.060 | 1.41–3.00 | **< 0.001** |
| Pathologic T stage (T2 vs. T1) | 1.840 | 1.33–2.53 | **< 0.001** | 1.740 | 1.26–2.40 | **0.001** |
| ER status (positive vs. negative) | 0.520 | 0.38–0.73 | **< 0.001** | 0.920 | 0.31–2.73 | 0.884 |
| PR status (positive vs. negative) | 0.500 | 0.37–0.69 | **< 0.001** | 0.670 | 0.39–1.15 | 0.143 |
| HER2 status (positive vs. negative) | 0.770 | 0.51–1.15 | 0.197 |  |  |  |
| Ki67% (>14 vs. ≤14) | 1.940 | 1.30–2.90 | **0.001** | 1.550 | 1.01–2.37 | **0.043** |
| Adjuvant hormonal therapy (yes vs. no) | 0.530 | 0.38–0.74 | **< 0.001** | 0.970 | 0.34–2.76 | 0.952 |
| Adjuvant chemotherapy (yes vs. no) | 1.330 | 0.94–1.89 | 0.112 |  |  |  |
| Adjuvant targeted therapy (yes vs. no) | 0.860 | 0.55–1.36 | 0.524 |  |  |  |
| BMI (overweight vs. normal weight) | 1.060 | 0.75–1.49 | 0.750 | 1.060 | 0.75–1.50 | 0.742 |
| BMI (obese vs. normal weight) | 1.890 | 1.18–3.02 | **0.008** | 1.740 | 1.08–2.79 | **0.022** |

Abbreviations: T, tumor size; ER, estrogen receptor; PR, progesterone receptor; HER2, human epidermal growth factor receptor 2; BMI, body mass index
